# 1658. Using remote Infectious Disease physician expertise to support inpatient antibiotic stewardship activities at three VA medical centers an implementation-effectiveness trial

**DOI:** 10.1093/ofid/ofac492.124

**Published:** 2022-12-15

**Authors:** Daniel J Livorsi, Stacey Hockett Sherlock, Cassie Cunningham Goedken, Kim Clarke, David Goodman, Sandra Pratt, Hyunkeun Cho, Heather Reisinger, Eli N Perencevich

**Affiliations:** University of Iowa Carver College of Medicine, Iowa City, Iowa; Iowa City VA Medical Center, Iowa City, Iowa; Iowa City VA Medical Center, Iowa City, Iowa; Carl VInson VA Medical Center, Dublin, Georgia; Bath VA Medical Center, Bath, New York; John J. Pershing VA Medical Center, Poplar Bluff, Missouri; University of Iowa Carver College of Medicine, Iowa City, Iowa; University of Iowa Carver College of Medicine, Iowa City, Iowa; University of Iowa/Iowa City VAMC, Iowa City, Iowa

## Abstract

**Background:**

Remote Infectious Disease (ID) physicians can provide stewardship support through telehealth. Using the RE-AIM framework, we assessed the implementation of telehealth-supported prospective-audit-and-feedback (tele-PAF) across 3 rural Veterans Administration medical centers (VAMC).

**Methods:**

All 3 invited sites agreed to participate and lacked ID support for stewardship at baseline. During 2021, an ID physician met virtually 3 times/week with the stewardship pharmacist champion at each participating VAMC to review patients on antibiotics in acute-care (mean daily census 3/site) and nursing-homes (NHs; mean census 71/site); real-time feedback on antibiotic use was given to clinicians. The primary outcome of effectiveness was monthly antibiotic days of therapy (DOT) per 1,000 days-present aggregated across all sites; the secondary outcome was days of antibiotic spectrum coverage (DASC) per 1,000 days-present. An interrupted time-series analysis was performed to asses these outcomes during the 1-year intervention period vs. the 2-year prior baseline. Semi-structured interviews with 20 clinicians and pharmacists were conducted to assess implementation.

**Results:**

RE-AIM elements are summarized in Table 1. Tele-PAF reviewed 502 unique patients and made 681 recommendations to 23 clinicians; 77% of recommendations were accepted. The most common recommendations were to stop antibiotics (28%) and change duration (20%). After the start of tele-PAF, antibiotic DOT and DASC immediately decreased in acute-care (-20%, p=0.01; -22%, p< 0.01) and NHs (-28%, p=0.03; -37%, p< 0.01). Both metrics began to rise again in acute-care (DOT: +2.5%/month, p=0.02; DASC: +2.7%/month, p=0.02) but were stable in NHs (Figure 1). Clinicians generally appreciated feedback, found it compatible with their workflow and responded favorably to collaborative discussions. Barriers included difficulty establishing rapport with some providers.

**Conclusion:**

The implementation of tele-PAF was associated with sustained reductions in antibiotic use across 3 NHs but not in the studied small acute-care units. Overall, clinicians perceived the intervention as acceptable and appropriate. Wider implementation of telehealth-supported stewardship activities may achieve reductions in antibiotic use.

**Disclosures:**

**Daniel J. Livorsi, MD**, Merck & Co.: Grant/Research Support.

